# Validation of the German Glasgow Sensory Questionnaire in autistic adults

**DOI:** 10.1186/s12888-025-06504-0

**Published:** 2025-01-31

**Authors:** Isabel Marie Jakob, Veit Roessner, Melanie Ring

**Affiliations:** 1 Department of Child and Adolescent Psychiatry, Medical Faculty, TUD Dresden, Technische Universität Dresden, Dresden, Germany; 2 Department of Child and Adolescent Psychiatry, Medical Faculty, Technische Universität Dresden, German Center for Child and Adolescent Health (DZKJ), partner site Leipzig/Dresden, Dresden, Germany

**Keywords:** Autism, Glasgow sensory questionnaire, Sensory sensitivity, Hypersensitivity, Hyposensitivity

## Abstract

**Background:**

We validated the German version of the Glasgow Sensory Questionnaire (GSQ), a self-report questionnaire for adults assessing the processing of stimuli regarding hypo- and hypersensitivity in seven sensory modalities. Since the GSQ is intended for the use in autistic adults, we aimed to complement our previous study on students with high and low Autism Spectrum Quotient (AQ; Zeisel et al., BMC Psychiatry 23:426, 2023), by surveying groups of autistic and non-autistic adults, to present the sensory processing profiles of the two groups, to identify the factor structure of the questionnaire in a group of autistic individuals and to identify the diagnostic value of a cut-off score for heightened sensory sensitivity in German autistic adults.

**Methods:**

A sample of autistic and non-autistic adults (each *n* = 86) completed the same German version of the GSQ as used in Zeisel et al. (BMC Psychiatry 23:426, 2023), the AQ and the Symptom Checklist-90-Revised. Factor analyses were applied.

**Results:**

The German GSQ showed good to excellent reliability. While the factor structure could not be confirmed, main findings of other validation studies were replicated: AQ and GSQ scores were moderately to strongly associated, with higher AQ and GSQ scores for autistic than for non-autistic individuals. Autistic individuals also showed more consistency in their sensitivity across sensory modalities. A third of the autistic participants had heightened sensory sensitivity, when a cut-off was set at the 95th percentile of the non-autism group. Conclusions: Overall, this German version of the GSQ can be considered a validated self-report questionnaire assessing sensory sensitivity particularly in autistic individuals. It can be used to assess sensory sensitivity in the diagnostic process of autism spectrum disorder and to assess an individual's sensory needs and strengths for best possible support. Further studies are required, especially to assess the internal structure of the GSQ.

**Supplementary Information:**

The online version contains supplementary material available at 10.1186/s12888-025-06504-0.

Autism spectrum disorder (ASD) is a lifelong neurological condition leading to experiencing the world differently. Part of the diagnostic criteria for ASD in the DSM-5 and ICD-11 and a very common phenomenon in autistic individuals [37], are sensory processing differences, like hyposensitivity, hypersensitivity and unusual interest in specific sensory stimuli [2, 64]. According to a model by Dunn [20], *hyposensitivity* includes poor registration—due to a high threshold for input of a certain sensory modality—and sensory seeking, a behavioral response to counteract the high threshold and to increase the level of and fulfill a need for sensory stimulation. *Hypersensitivity* includes sensitivity to stimuli—due to a low threshold for sensory input of a certain modality—and sensory avoiding, a behavioral response to counteract the low threshold and reduce sensory input. In addition, individuals` responses do not necessarily need to counteract the threshold, they could also go in accordance with the threshold, e.g. individuals with a high sensory threshold might respond to few stimuli and vice versa for individuals with a low threshold [20].

Self- and parent-report studies have found that autism-like traits correlate with unusual sensory processing in autistic and non-autistic individuals [39] and that autistic individuals show more hyposensitivity, hypersensitivity, sensory seeking and sensory avoidance than non-autistic individuals [22, 30, 31]. The prevalence for sensory atypicalities in autistic adults is reported between 70% [5] to 95% [16, 37]. Qualitative research suggests that many autistic adults experience multiple sensory atypicalities although hypersensitivity seems to be more common than hyposensitivity [37]. However, while some sensory atypicalities might be similarly severe, there seems to be great variability in sensory atypicalities among autistic individuals [16]. Sensory atypicalities can affect all sensory modalities. Many autistic adults seem to experience *visual* hypersensitivity for bright or flashing lights [37]. Qualitative as well as experimental studies found that autistic adults showed hypersensitivity towards *auditory* stimuli like loud noises or different noises at the same time as well as sensory avoidance in regard to those stimuli [28, 37]. Autism seems to go along with hypersensitivity to strong odors [37], however, experimental studies and meta-analytic studies found that odor [8, 17], meta-analysis [25, 34, 62], and *taste* identification appear to be impaired [8, 54]. Experimental studies had conflicting results as to whether autism seems to be associated with heightened [3] or decreased *olfactory* perception [34]. Qualitative as well experimental research reported *tactile* hypersensitivity in autistic individuals to specific textures or pieces of clothing [37] and to being touched [9, 47], where touch was experienced as less pleasant or even unpleasant [14]. Self-report research reported that autistic individuals show sensory avoiding as well as sensory seeking behavior towards *vestibular* sensory events [30, 31]. To conclude, most of these previous studies included relatively small samples (*n* < 56, two studies with *n* = 103).

As the sensory atypicalities experienced by autistic individuals can vary wildly, contradicting study results regarding an enhanced or reduced performance are not surprising. Null results could even be explained by a balance between the included autistic individuals that experience hypersensitivity to the specific sensory modality and those that experience hyposensitivity. Therefore, it is urgently necessary to conduct studies with larger samples using manageable and economical instruments for valid measurement of sensory atypicalities.

These instruments, for instance the Glasgow Sensory Questionnaire (GSQ, [48]), are also necessary in the diagnostic process of ASD, as experiencing hypo- and/or hypersensitivity is a diagnostic criterion and of clinical interest to assess an individual's specific sensory atypicalities. Gaining more insight into the sensory processing of autistic individuals could furthermore promote scientific research regarding the underlying mechanisms of autism and give us a foundation on how to create more sensory-friendly environments for autistic individuals. The GSQ is a self-report questionnaire for adults assessing sensory processing regarding hypo- and hypersensitivity (domains) in the seven sensory modalities visual, auditory, gustatory, olfactory, tactile, vestibular and proprioception [48]. It was designed based on reports of sensory processing by autistic individuals (or their caregivers; [48]). A high correlation between autism-like traits, measured by the Autism-Spectrum Quotient (AQ, [6]), and sensory sensitivity, measured by the GSQ, was found [48]. The GSQ has been translated to and validated in French [50], Dutch [35], Japanese [53, 58], *n* = 95 psychology student subjects), Chinese [60], *n* = 531 general population subjects) and German [65]. The results by Robertson and Simmons [48] in a general population sample (*n* = 212) were replicated in the Japanese validation study in a sample of autistic and non-autistic adults [53], *n* = 64 autistic subjects). The Dutch validation study assessed test–retest stability over 12 weeks, finding that the GSQ was stable over time [35], *n* = 78 autistic subjects). Kuiper et al. [35], furthermore examined an aspect of clinical use for the GSQ. They determined a cut-off for *heightened sensory sensitivity* at the 95th percentile of the non-autism group and found that almost 65% of the autistic adults in this study had a score above that cut-off and, therefore, showed heightened sensory sensitivity [35]. Sapey-Triomphe et al. ([50], *n* = 95 autistic subjects) assessed the structure of the questionnaire and found the GSQ items to load onto two factors, one factor mostly grouping hyposensitivity and the second factor mostly grouping hypersensitivity items. Furthermore, they examined sensory profiles, looking at differences between individuals with high and low AQ scores and inferred from their findings, that autistic individuals showed a greater consistency in their sensitivity across sensory domains [50]. Zeisel et al. [65] validated a German version of the GSQ in a student sample (*n* = 297). Reliability was satisfactory, AQ and GSQ scores were associated and high AQ students had higher GSQ scores than low AQ students [65]. However, group differences did not persist for all modalities and neither the factor structure of Sapey-Triomphe et al. [50] nor their sensory profiles could be replicated [65]. One possible reason might be the small number of high AQ students (*n* = 35) included in that study. In addition, the GSQ was originally designed for the use in autistic individuals. Therefore, the current study complements this previous one by examining the validation of the same German version of the GSQ in autistic compared to non-autistic individuals and characterizing their sensory profiles.

Hypotheses are based on the expectation of a successful validation. We expected the German version of the GSQ to show at least acceptable reliability. Furthermore, we expected autistic individuals to show higher AQ scores and experience higher levels of sensory sensitivity. We expected a positive correlation between AQ scores and sensory sensitivity and between hypo- and hypersensitivity. Lastly, we expected the German GSQ to show a comparable internal structure to the original study (one factor). In relation to Sapey-Triomphe et al. [50] and Kuiper et al. [35], we explored the sensory profiles and the diagnostic capabilities of the German GSQ by inspecting a cut-off score for heightened sensory sensitivity for autistic adults.

## Methods

### Participants

As part of a larger study 86 autistic and 87 non-autistic participants were recruited between May 2020 and May 2021. Autistic individuals were contacted through the autism centre of the University Hospital Carl Gustav Carus Dresden and asked to participate. They had a prior clinical diagnosis of either Childhood Autism (F84.0, 55.8%), Atypical Autism (F84.1, 1.2%) or Asperger Syndrome (F84.5, 43%) according to the ICD-10 [63] and were diagnosed mostly using the diagnostic gold standard for autism i.e., the Autism Diagnostic Interview-Revised (ADI-R [11]) and the Autism Diagnostic Observation Schedule (ADOS [45, 49]). Autistic individuals had an Intelligence Quotient (IQ) of at least 75 and were able to answer the questions themselves. IQ had been assessed previously by the German Cultural Fair Intelligence Test Scale I [61] or the Wechsler Intelligence Scale for adults [43, 55, 59] or children [44, 56]. Autistic participants with co-occurring diagnoses were not excluded from the study (43.0% had one, 11.6% had two, 19.8% had three or more co-occurring mental or behavioral disorders). Non-autistic individuals were recruited from the general population through advertisements on Facebook and Ebay as well as from a hospital database of research participants through e-mail and phone calls. They answered screening questions and were only included if they reported no neurological, developmental or psychiatric diagnoses in their personal or family history as well as no heightened use of illegal substances (using cannabis more than five or other illegal substances more than two times a year). One individual of the non-autism group was excluded from the data analyses because they had an AQ score of 36, which is above the suggested cut-off score of 32 on this instrument for the non-autism group [6]. No individual had to be excluded because of missing data. The final sample consisted of *n* = 86 participants in the autism group (75.6% male; age range: 18 – 67 years; age of diagnosis: 4 – 59 years) and *n* = 86 participants in the non-autism group (76.7% male; age range: 18 – 70 years, descriptive data in Table 1).

**Table 1:** Descriptive data for the autism and the non-autism group

	Autism Group (65 male, 21 female)		Non-autism Group (66 male, 20 female)	
	*M*	*SD*	*M*	*SD*
Age (in years)	33.5	13.3	34.5	14.2
SCL-90-R, GSI T-value^a^	59.7	9.1	52.6	11.0
Full-Scale IQ	103.8	16.0		
Age of diagnosis	24.3	15.5		
ADOS-2 (*n* = 79-83)^b^				
- combined com. / soc. int.	11.8	4.2		
- subscale communication	4.1	1.8		
- subscale social interaction	7.8	2.7		
- subscale creativity	1. 2	0.70		
- subscale stereotyped behaviors, restricted interests	1.1	1.1		
ADI-R (*n* = 67-68)^c^				
- subscale social interaction	16.7	5.6		
- subscale communication	11.6	4.2		
- subscale repetitive behavior	4.0	2.6		
Education	*n*	%	*n*	%
- no degree	4	4.7	0	0.0
- degree after 9 years	5	5.8	5	5.8
- degree after 10 years	37	43	27	31.4
- high school degree	17	19.8	28	32.6
- university degree	19	22.1	24	27.9
- postgraduate degree	4	4.7	2	2.3

Information on ethnicity and socioeconomic status was not recorded

^*a*^ T-value of the Global Severity Index (GSI) score of the Symptom Checklist-90-Revised (SCL-90-R, [18])

^b^ Not all participants were diagnosed at the autism centre of the University Hospital Carl Gustav Carus Dresden, therefore, for a few participants ADOS-2 scores were not available

^c^ Some participants were diagnosed with a thorough review of developmental history but without using the ADI-R as no parent or other informant was available

The groups were comparable regarding age, *t*(170) = −0.48, *p* = .63, *d* = −0.07, gender, χ^2^(1, *N* = 172) = 0.03, *p* = .86, φ = 0.01, and education, *U* = 3187.00, *Z* = −1.64, *p* = .10, *r* = −0.12. However, autistic individuals reported more psychological distress as assessed by the Global Severity Index (GSI) of the SCL-90-R [18] than non-autistic individuals, *t*(158.3) = 4.52, *p* < .001, *d* = 0.70, with almost 50% experiencing heightened levels of psychological distress (T ≥ 60) compared to about 30% in the non-autism group. Psychological distress was assessed because the study took place during the Covid-19 pandemic.

### Procedure

This study was part of a larger project investigating anxiety in autism including previous publications by Zeisel et al. [65], Riedelbauch et al. [46] and Thiel et al. [57]. It was approved by the ethics committee of the TU Dresden (ethical approval code: EK 356092018) and carried out in accordance with these regulations and the declaration of Helsinki. All participants and when applicable in the case of autistic participants their guardians gave written informed consent. The larger study consisted of twelve questionnaires, whose completion took about 60 to 90 min and of which the German AQ [24], the German version of the GSQ [48] and the Symptom Checklist-90-Revised (SCL-90-R, [19]) are relevant here. Participants filled in the questionnaires at home either on paper or online on LimeSurvey (Version 2.72.3, [36]). They received a compensation of 10€ after completion.

### Measures

The *Autism Spectrum Quotient* (AQ, [6], German version by [24]) is a validated 50-item self-report questionnaire assessing autism-like traits in adults. There are five subscales, social skill, attention switching, attention to detail, communication and imagination, with 10 items each. Items are statements to which respondents indicate the degree of their agreement on a 4-point Likert scale (strongly disagree to strongly agree), however, scoring is dichotomous. A total score between 0 and 50 can be received, with a higher score indicating more autism-like traits. The AQ is considered a screening tool for ASD, for which a cut-off score of 26 is suggested, and can differentiate between non-autistic and autistic individuals [6]. However, non-autistic individuals with social anxiety disorder (SAD) or obsessive–compulsive disorder (OCD) have been found to have higher AQ scores than non-autistic healthy individuals, which may be explained by a symptom overlap between autism and some psychiatric disorders [15]. As only a very small percentage of non-autistic individuals score above 31 [6], a cut-off score of 32 is appropriate, when using the AQ score as an exclusion criterion for a non-autism group.

The *Glasgow Sensory Questionnaire* (GSQ, [48]) is a validated 42-item self-report questionnaire assessing sensory sensitivity in the general population as well as in autistic adults. The GSQ consists of two domains and seven modalities. Overall, there are 14 subscales, one for each combination of modality and domain, with three items each. Participants indicate how often they experience the in the item described situation or phenomenon on a 5-point Likert scale (never, rarely, sometimes, often, always). Responses are scored from 0 (never) to 4 (always). A total score between 0 and 168 can be received, with higher scores indicating more sensory sensitivity. For the purpose of our larger project, we translated and back-translated the original English version to German with permission of the authors (Robertson & Simmons, University of Glasgow). Therefore, the German translation used in this study is the same as used and presented in Zeisel et al. [65].

The *Symptom Checklist-90-Revised* (SCL-90-R, [18],German version by [33]) is a validated 90-item self-report questionnaire assessing psychological distress of the previous week. Amongst other dimensions, symptoms regarding obsessive–compulsive behavior, depression and anxiety are asked about. Items are answered based on how bothered the respondent was by the described symptom. There are three global scores including the Global Severity Index (GSI), which summarizes the intensity of perceived psychological distress of all items. Raw scores can be transformed into normed T-values, with T-values of 60 or more indicating heightened psychological distress [23, 33]. An association between psychological distress and sensory sensitivity was shown previously [4, 7].

### Statistical analysis

Hypotheses, statistical analyses and exclusion criteria as well as how to deal with missing data were preregistered on OSF after completion of the study, but before the data were viewed. Sample size calculations were done using G*Power [21].

For individuals with missing data (*n* = 9), AQ total and subscale and GSQ total and domain scores were corrected by adding the mean item score. Reliability was assessed with Cronbach's alpha. Between group differences regarding AQ and GSQ scores were assessed with independent sample *t-*tests. An exploratory three-way mixed analysis of variance (ANOVA) was done to explore interaction effects (Greenhouse–Geisser correction used because of violations of sphericity). Relationships between AQ and GSQ scores and between the domains hypo- and hypersensitivity were assessed using Pearson correlation analysis. Post-hoc partial correlation analysis was used to control for psychological distress. Group differences regarding the correlations between AQ and GSQ and between hyposensitivity and hypersensitivity across the sensory modalities were explored using Fisher r-to-Z transformation. The internal structure of the German GSQ was assessed with unweighted least squares (ULS) and maximum likelihood (ML) confirmatory factor analyses (CFA). Fit indices were interpreted according to Hooper et al. [26]. For that, participants with any missing data in the GSQ were excluded (*n* = 6 from the autism, *n* = 3 from the non-autism group, difference between groups not significant, χ^2^(1, *N* = 172) = 1.01, *p* = .3, φ = −0.08). In search of a better fitting model, further factor analyses were tested in an exploratory manner. To explore the diagnostic capabilities of the GSQ, a cut-off score for heightened sensory sensitivity levels was calculated (95th percentile of GSQ total score of the non-autism group). Further analyses regarding reliability, a comparison with the original study by Robertson & Simmons [48], effects of sex and age on sensory sensitivity, correlations between AQ and GSQ on a subscale level, associations between GSQ subscales and further exploratory tested factor analyses as well as further tables and figures depicting results of the analyses described below can be found in the supplementary material (see SupplementaryMaterial.pdf).

A *p*-value of 0.05 was used as level of significance for all analyses. Bonferroni-Holm correction was applied for multiple comparisons or correlations. Effect sizes are reported. Most statistical analyses were conducted using IBM SPSS Statistics (28.0.0.0) and IBM SPSS AMOS (28.0.0.0). Python (3.10.09) was used to create all figures.

## Results

### Reliability of the German GSQ

Cronbach's Alpha of the German GSQ on an item level was 0.92 in the entire sample (*N* = 163), 0.93 in the autism group (*n* = 80) and 0.86 in the non-autism group (*n* = 83). Mean of the corrected item-total correlations (CITC) was 0.45, 0.46 and 0.34 respectively, with CITCs below 0.3 for the Items 17, 22, 28, 36 and 39 in the entire sample, 17, 36 and 39 in the autism group and for 20 items in the non-autism group (correlations below 0.2 for the Items 3, 22 and 28). Cronbach's Alpha on a subscale level was 0.90 in the entire sample (*N* = 163), 0.91 in the autism group (*n* = 80) and 0.83 in the non-autism group (*n* = 83), with a mean of the corrected subscale-total correlations (CSTC) of 0.60, 0.61 and 0.47 respectively. There were no CSTCs below 0.3 in either group or the entire sample.

### Group comparison

#### AQ

Total and all subscale AQ scores were higher in the autism than in the non-autism group (see Table 2).

**Table 2:** AQ scores in the autism and non-autism group

	Autism Group (*n* = 86)		Non-autism Group (*n *= 86)		Comparison			
	*M*	*SD*	*M*	*SD*	*t*	df	*p*	*d*
AQ total score	32.8	8.9	17.3	6.9	12.86	156.0	< .001	1.96
AQ subscale scores								
- social skill	7.1	2.6	2.8	2.5	10.92	170	< .001	1.67
- attention switch.^a^	7.7	2.2	4.2	2.1	10.52	170	< .001	1.61
- attention to detail	5.9	2.5	4.5	2.5	3.72	170	< .001	0.57
- communication	6.1	2.7	2.0	1.8	11.52	146.0	< .001	1.76
- imagination	6.0	2.2	3.7	1.9	7.34	170	< .001	1.12

*p*-values for subscale comparisons Bonferroni-Holm corrected

^a^ subscale attention switching

#### GSQ

An exploratory 2 (*group* [autism, non-autism]) × 2 (*domain* [hypo, hyper]) × 7 (*modality* [visual, auditory, gustatory, olfactory, tactile, vestibular, proprioception]) mixed ANOVA was calculated (homogeneity of error variances, Levene's Test, *p* < .04 for all except gustatory / olfactory hypo, and of covariances, Box's test, *p* < .001, was not given). There was a main effect for *group*, *F*(1, 161) = 30.01 (GG), *p* < .001, η^2^ = 0.16. GSQ total, domain and modality scores were higher in the autism group than in the non-autism group (see Table 3). Autistic individuals scored higher than non-autistic individuals in 10 out of the 14 GSQ subscales (exploratory comparison, *p* between < .001 and .01, *d* between 0.48 and 0.96; no significant differences in the subscales visual / gustatory / olfactory hypo, and tactile hyper, *p* between .06 and .72, *d* between −0.06 and 0.38; *p*-values corrected).

**Table 3:** GSQ scores in the autism and non-autism group

	Autism Group (*n* = 86)		Non-autism Group (*n *= 86)		Comparison			
	*M*	*SD*	*M*	*SD*	*t*	df	*p*	*d*
GSQ total score	55.1	24.3	38.3	14.8	5.49	140.1	< .001	0.84
Domain scores								
- hyposensitivity	26.9	11.3	19.4	7.6	5.14	149.0	< .001	0.78
- hypersensitivity	28.2	14.4	18.9	8.8	5.10	140.6	< .001	0.78
Modality scores ^a^								
- visual	7.1	4.7	5.1	3.3	3.31	151.5	.001	0.51
- auditory	12.9	5.0	9.5	3.9	4.92	158.3	< .001	0.75
- gustatory	6.4	3.7	4.5	2.7	3.91	156.2	< .001	0.60
- olfactory	6.5	4.4	5.3	2.8	2.09	140.5	.02	0.32
- tactile	8.4	4.3	6.4	2.8	3.63	145.5	< .001	0.55
- vestibular	7.5	4.6	3.9	2.8	6.28	140.8	< .001	0.96
- proprioception	6.2	3.8	3.6	2.6	5.13	148.1	< .001	0.79

Comparisons calculated with one-sided independent sample* t*-tests*. p*-values for domain and modality comparisons Bonferroni-Holm corrected

^a^ Sample size for each modality and group between *n* = 84 and *n* = 86 due to missing answers (see Table 4 for specific sample sizes)

**Table 4:** Associations between the domains across the sensory modalities

	Autism Group	Non-autism Group	Group differences
Visual	.57*** (*n* = 85)	.39** (*n* = 85)	*z* = 1.51 *p* = .66
Auditory	.61*** (*n* = 85)	.59*** (*n* = 86)	*z* = 0.20 *p* > .99
Gustatory	.27* (*n* = 85)	.30* (*n* = 86)	*z* = −0.21 *p* > .99
Olfactory	.46*** (*n* = 84)	.04 (*n* = 86)	*z* = 2.93 *p* = .02*
Tactile	.47*** (*n* = 86)	.14 (*n* = 86)	*z* = 2.38 *p* = .10
Vestibular	.29* (*n* = 86)	.26* (*n* = 85)	*z* = 0.21 *p* > .99
Proprioception	.49*** (*n* = 84)	.40** (*n* = 85)	*z* = 0.72 *p* > .99

Two-tailed. Bonferroni-Holm corrected applied to all *p*-values

^*^significant on a .05 level

^**^significant on a .01 level

^***^significant on a .001 level

**Fig. 1:**
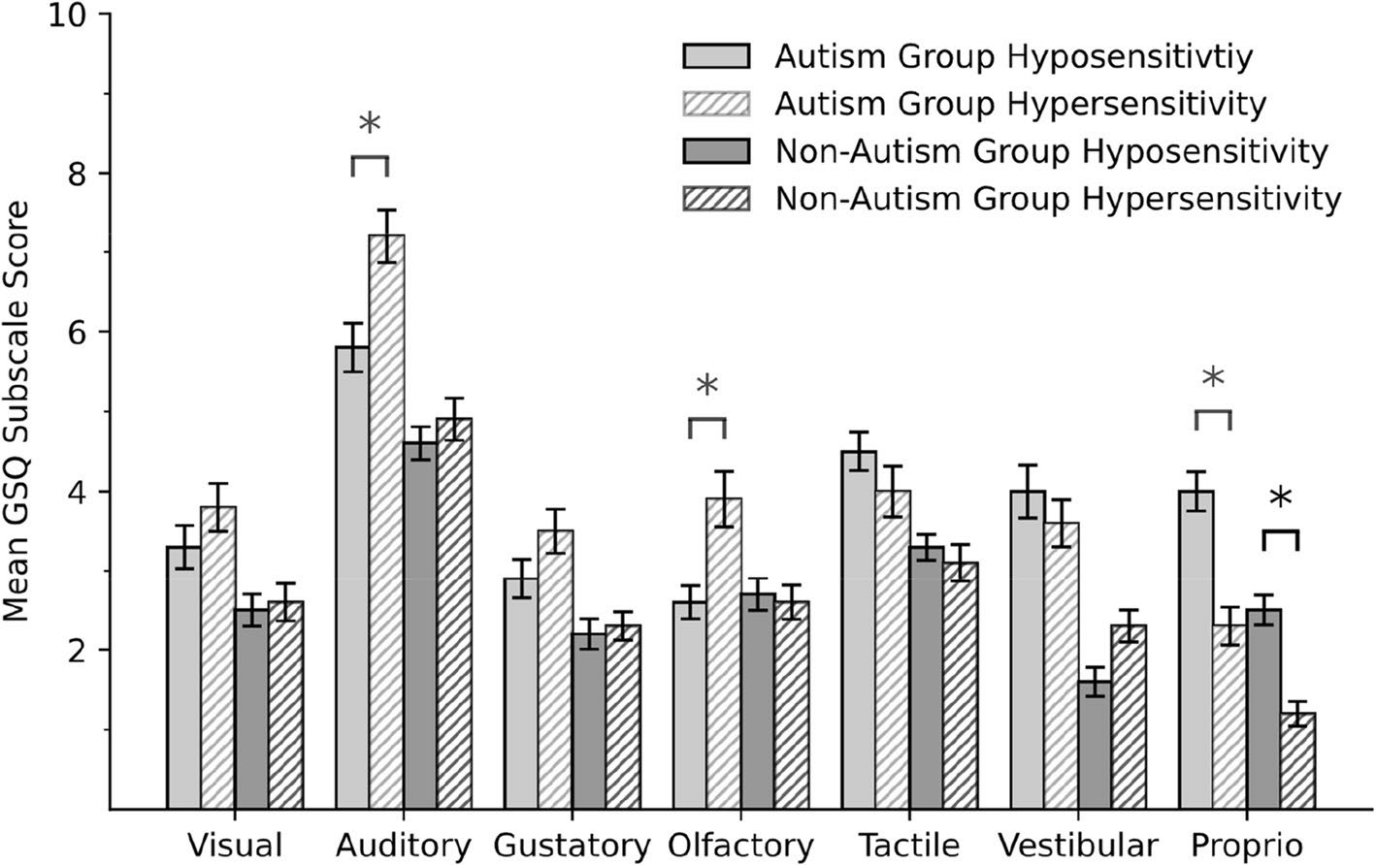
GSQ modality scores for the domains hyposensitivity and hypersensitivity in the autism and the non-autism group (interaction effect group x modality x domain). *Note.* Depicted are the mean scores of each modality in the domains hypo- and hypersensitivity separated by group. Error bars show the standard errors. Proprio = Proprioception. * significant differences between the domains on a .05 level, *p*-values Bonferroni-Holm corrected

There was no *domain* main effect, *F*(1, 161) = 1.15 (GG), *p* = .3, η^2^ = 0.01, but a *modality* main effect, *F*(5.1, 823.5) = 110.42 (GG), *p* < .001, η^2^ = 0.41. Post-hoc tests showed, more sensory sensitivity in the auditory domain than in any other domain (*p*-values Bonferroni-Holm corrected). There was a *group* x *modality* interaction effect, *F*(5.1, 823.5) = 3.67 (GG), *p* = .002, η^2^ = 0.02. While the autism group scored higher in all modalities, the size of that effect differed between modalities. There was a *domain* x *modality* interaction effect, *F*(5.6, 161) = 20.78 (GG), *p* < .001, η^2^ = 11, as well as a *group* x *domain* x *modality* interaction effect, *F*(5.6, 161) = 5.45 (GG), *p* < .001, η^2^ = 0.031 (see Fig. 1).

### Association between AQ and GSQ

**Fig. 2:**
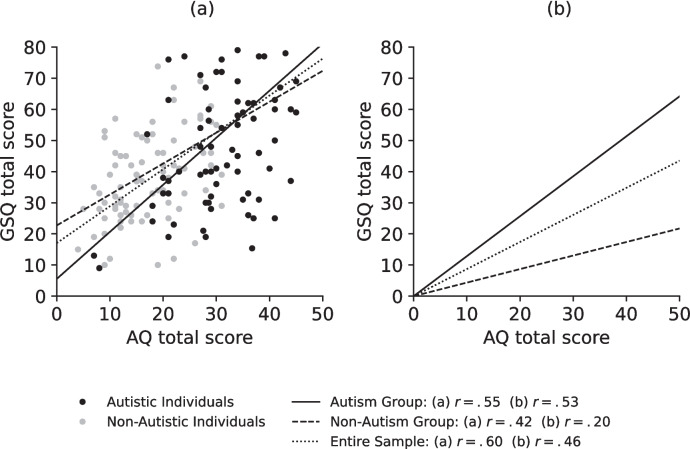
Correlation between AQ and GSQ total scores*. Note*. **a** Correlation between AQ and GSQ total scores. **b** Correlation between AQ and GSQ total scores controlled for psychological distress

There was a strong positive correlation between AQ and GSQ total scores in the entire sample, *r* = 0.60, *p* < .001, and the autism group, *r* = 0.55, *p* < .001, and a moderate correlation in the non-autism group, *r* = 0.42, *p* < .001 (see Fig. 2(a)). As the groups differed in perceived psychological distress, post-hoc analyses were done. Partial correlation analysis revealed a moderate correlation between AQ and GSQ in the entire sample when controlling for the SCL-90-R GSI T-value (*N* = 160), *r* = 0.46, *p* < .001, a strong correlation in the autism group, *r* = 0.53, *p* < .001, and only a small positive correlation in the non-autism group, *r* = 0.20, *p* = .03 (see Fig. 2(b)). Exploratory analysis revealed a difference in the associations between AQ and GSQ total scores between the autism and the non-autism group, when controlling for psychological distress,* z* = 2.40, *p* = .02. However, there was no difference between the associations without controlling for perceived psychological distress, *z* = 1.10, *p* = .27.

### Associations between GSQ subscales and domains

There were strong positive correlations between the GSQ hypo- and hypersensitivity scores in the entire sample, *r* = 0.77, *p* < .001, the autism, *r* = 0.79, *p* < .001, and the non-autism group, *r* = 0.62, *p* < .001. The correlation between hypo- and hypersensitivity was greater in the autism group than in the non-autism group, *z* = 2.23, *p* = .03 (exploratory). There were correlations between the hypo- and hypersensitivity subscale scores for all sensory modalities in the autism group and for five out of the seven modalities in the non-autism group (Table 4). There was only a difference between the groups in the olfactory modality, *z* = 2.93, *p* = .02, with a greater correlation in the autism than in the non-autism group (exploratory analysis).

To assess sensory profiles, correlations between all 14 GSQ subscales separated by group were calculated (see Table 5). Out of the 91 correlations, 68 were significant in the autism group compared to 26 correlations in the non-autism group. There were stronger correlations in the autism group (*M*_*r*_ = 0.42, *SD*_*r*_ = 0.11) than in the non-autism group (*M*_*r*_ = 0.27, *SD*_*r*_ = 0.13), *t*(171.9) = 8.12, *p* < .001, *d* = 1.20. Congruently to Sapey-Triomphe et al. [50] and contrary to Kuiper et al. [35], there were stronger correlations between the GSQ subscale scores (correlation matrix) in the autism group than in the non-autism group.

**Table 5:**
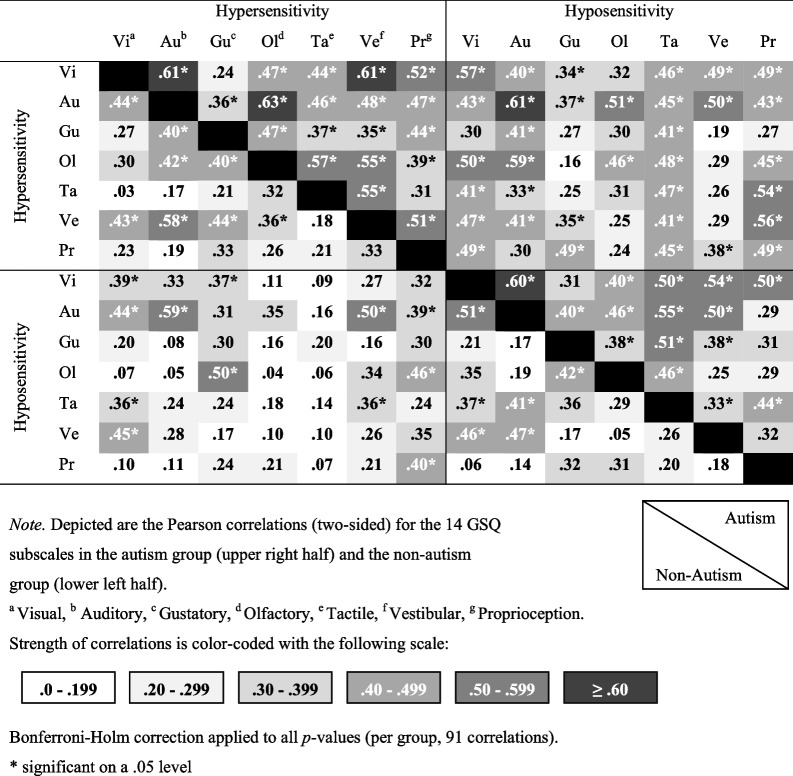
Correlation matrix for the 14 GSQ subscales in the autism group and the non-autism group

### Factor structure of the German GSQ

Outliers, as indicated by Mahalaboni distance with *p* < 0.001, were calculated for the entire sample (*n* = 4) and for the autism and non-autism groups (both *n* = 0) and were excluded in the corresponding CFAs. Kaiser–Meyer–Olkin measure of sampling adequacy had a value of 0.83 for the entire sample, 0.71 for the autism and 0.57 for the non-autism group. Bartlett's test of sphericity was < 0.001 for the entire sample and both groups, indicating that data were appropriate for factor analysis [29].

CFAs with ULS and ML of a single factor model aggregating all 42 items into the factor sensory sensitivity were calculated for the entire sample and both groups. As standardized regression weights did not differ substantially between ULS and ML, only ULS results are reported. The 42 items had a mean standardized regression weight of 0.46 in the entire sample (0.05—0.72, five items below 0.3), 0.48 in the autism group (0.02—0.80, three items below 0.3) and 0.35 in the non-autism group (−0.003—0.61, 16 items below 0.3).

Fit indices are shown in Table 6 and interpreted according to Hooper et al. [26]. Results were mixed, but overall, the model did not seem to fit very well. Chi statistics were significant, which they should not have been, and comparative fit indices (CFI) were well below 0.9, which they should at least have been. The normed chi-square values, however, were in the acceptable range, with suggestions between < 2 and < 5. For the goodness-of-fit index (GFI), a more conservative recommendation of > 0.95 and a more liberal recommendation of > 0.9 exists. When applying the liberal cut-off an acceptable fit of the model in the entire sample and the autism group was indicated by the GFI. The root mean square error of approximation (RMSEA) was scraping on the acceptable fit range in the entire sample (Lo90 = 0.069, Hi90 = 0.08), with suggestions of < 0.07 and an upper limit of < 0.08 for an acceptable fit. [26].

**Table 6:** Fit indices of the confirmatory factor analyses

	χ^2^	df	*p*	χ^2^/*df*^*a*^	GFI^b^	CFI^c^	RMSEA^d^
Entire Sample							
- ULS^e^	1019.7	819		1.25	.93		
- ML^f^	1540.5	819	*< *.001	1.89		.65	.075*
Autism Group							
- ULS	1140.0	819		1.39	.91		
- ML	1409.5	819	*< *.001	1.72		.56	.096*
Non-Autism Group							
- ULS	564.8	819		0.69	.81		
- ML	1351.9	819	< .001	1.65		.40	.089*

Entire Sample: *N = *159, *n* = 76 autism group, *n* = 83 non-autism group (difference not sign., χ^ 2^(1, *N* = 159) = 0.31, *p = *.58, φ = 0.04); Autism Group: *n = *80; Non-Autism Group: *n = *83

^a^ relative / normed chi-square, ^b^ Goodness-of-fit index, ^c^ Comparative fit index, ^d^ Root mean square error of approximation, ^e^ Unweighted least squares method, ^f^ Maximum Likelihood method

* significant on a .05 level

Further exploratory analyses were done to see if model fit could be improved. When excluding items from the factor model, that according to item-total correlations and regression weights did not seem suitable [41], there was a slightly improved model fit in the non-autism group. A second-order model with the domains hypo- and hypersensitivity each grouping the 21 items belonging to them and the factor sensory sensitivity over the domains did not improve model fit. A third-order model, as tested in Zeisel et al. [65], with the subscales grouping their three items each and the domains grouping their subscales and the overarching factor sensory sensitivity, improved model fit in the entire sample and the autism group slightly. However, a good model fit could not be achieved with either model.

Finally, we used exploratory factor analyses to investigate the factor structure further. For the entire sample, six factors were extracted (χ^2^ (624) = 2712.4, Tucker Lewis Index = 0.874, RSMEA = 0.039), for the non-autism sample four (χ^2^ (699) = 1445.17, Tucker Lewis Index = 0.809, RSMEA = 0.035) and for the autism sample one (χ^2^ (819) = 1797.88, Tucker Lewis Index = 0.64, RSMEA = 0.068). While we could not find a sensual interpretation for the factor solutions for the entire sample and the non-autism group, the one-factor-solution of the autism group replicated the original structure of the questionnaire [48].

### Heightened sensory sensitivity (exploratory)

**Fig. 3:**
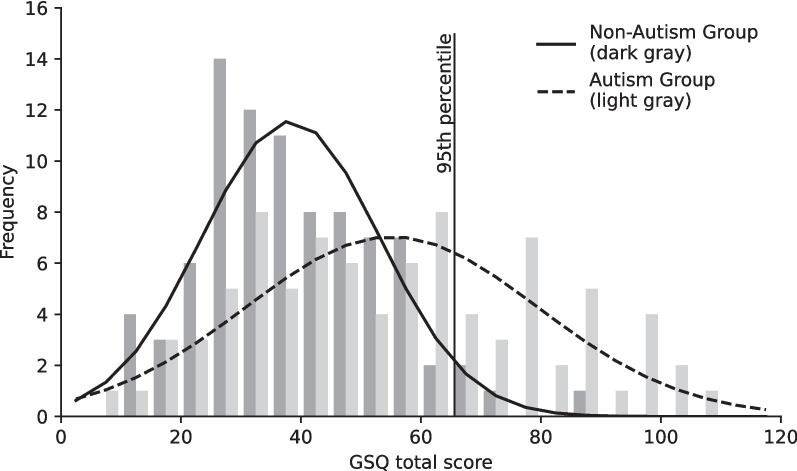
Histogram of GSQ total scores by group including the 95th percentile of the non-autism group

The 95th percentile for the GSQ total score of the non-autism group was 65.6, with only 4/86 non-autistic participants scoring higher. When appointing 65.6 as the cut-off score, about one third of the autistic participants (33.7%, 29/86) reported *heightened sensory sensitivity levels* (see Fig. 3). For comparison the 90th and 99th percentiles were calculated. When dropping the cut-off score to the 90th percentile of the non-autism group (GSQ score of 57), 47.7% of autistic participants scored at or above the cut-off. When raising the cut-off score to the 99th percentile of the non-autism group (GSQ score of 74), 25.6% of autistic participants scored higher still.

## Discussion

We validated a German version of the GSQ in a larger and well-matched sample of autistic and non-autistic adults and characterized their sensory profiles, complementing the previous validation study of this German GSQ in a student sample by Zeisel et al. [65]. Reliability as assessed by Cronbach’s alpha was good to excellent and slightly better in the autism group. Results were very similar to the original British study by Robertson & Simmons [48], as well as the Dutch, French, Japanese and Chinese validation studies [35, 50, 53, 60]. Cronbach’s alpha as well as mean CITCs were slightly higher in this sample than in the student sample of Zeisel et al. [65], with their mean CITC of 0.36 more closely resembling that of our non-autism group (0.34). There were a few problematic items (Items 17, 22, 28, 36 and 39). The Items 17 and 36, both assessing olfactory hyposensitivity, as well as Item 22, assessing tactile hypersensitivity, have also been found unsatisfactory in previous studies [50, 58, 65]. The very small negative CITC of Item 35 in the high AQ group of Zeisel et al. [65], was not found here, suggesting that the item is valid, and the finding of Zeisel et al. [65] is not explained by translational or cultural reasons but was coincidental due to the small subsample size.

As expected, there was more hypo- as well as hypersensitivity reported by the autism than the non-autism group, however, that difference was smaller than in the Dutch or French validation studies [35, 50]. Autistic individuals reported significantly more sensory sensitivity than non-autistic individuals in all sensory modalities, which is in line with previous studies, but differs from Zeisel et al. [65] where the high and low AQ group only differed in the auditory and tactile modalities. This strengthens the idea that autistic individuals differ in their sensory sensitivity from non-autistic individuals with similar AQ scores and that high AQ scores should not be used as a proxy for ASD. However, in this study too not all subscales differed between groups (10/14 did). Olfactory hyposensitivity was even higher in the non-autism group (though not significantly). This is in line with previous findings of autistic individuals showing olfactory hypersensitivity [3, 37], however, contradicts the findings of decreased olfactory perception [34]. In line with previous research were the findings, that autistic individuals only showed more visual hyper- but not hyposensitivity [37] and that they showed more vestibular hypo- as well as hypersensitivity [30, 31]. While previous research suggested primarily hypersensitivity in the auditory modality [28, 32, 37] and hyposensitivity regarding proprioception [10, 47], in the current study, autistic participants reported more hypo- as well as hypersensitivity than non-autistic participants in both domains. Regarding the gustatory modality, hypo- as well as hypersensitivity was found previously [8, 37, 54], but here autistic participants only reported an increased gustatory hypersensitivity. Contrary to previous findings, which suggested tactile hypersensitivity in autistic individuals [9, 47], autistic participants only reported more tactile hyposensitivity than non-autistic participants. An ANOVA revealed that there was a main effect of modality, just as in Zeisel et al. [65], with higher scores in the auditory modality than in any other in this sample, which was also found in the original British study [48], suggesting that auditory hypo- and hypersensitivity is more common in autistic as well as non-autistic individuals than sensitivity to any other sensory modality. In line with Zeisel et al. [65] and in contrast to Sapey-Triomphe et al. [50], who found higher hyper- than hyposensitivity scores, there was no main effect of domain. This might be attributed to the sample period during the first year of Covid-19 with the possible explanation that autistic as well as non-autistic individuals might have experienced fewer of the situations described in the GSQ especially regarding hypersensitivity or were less bothered by them prior to answering the questionnaire as people might have been staying more in the comfort of their own home. There is not a lot of research on sensory sensitivities during the COVID-19 pandemic, however, our argument is supported by study from India showing that sensory sensitivity in autistic children mostly improved or stayed the same during the pandemic, whereas it increased in only a third of the sample researched by parent interview ([38],but see [12] showing an increase in sensory difficulties in autistic children). It remains to be seen whether this result stays consistent in further studies. What has been consistent throughout the studies, however, were higher hypo- than hypersensitivity scores in the proprioception modality in both groups [50, 65]. While there were no group interaction effects found in a student sample [65], in this sample interaction effects between group and modality as well as group, domain and modality were found, which could be attributed to the small sample size in Zeisel et al. [65] or further suggest that while mean GSQ and AQ scores between the groups of Zeisel et al. [65] and this study were similar, sensory atypicalities of the autism group of this study differed from the high AQ group of Zeisel et al. [65].

The moderate to strong correlations found between autism-like traits and sensory sensitivity are in line with previous validation studies [35, 50, 53, 65]. However, this correlation vanished in the non-autism group but not in the autism group, when controlling for perceived psychological distress. A remotely similar result was found in a previous study in which the correlation between AQ and GSQ decreased from 0.48 to 0.35 when controlling for anxiety [27]. This could possibly be explained by a symptom overlap between autism and some psychiatric disorders such as SAD or OCD (i.e. [15, 52]). Non-autistic individuals with SAD or OCD have been found to have higher AQ scores than non-autistic healthy individuals [15]. While participants in our non-autism group did not have or were not aware of having any psychological disorders, symptoms of those which they reported in the SCL-90-R could still have been reported in the AQ as well. As psychological distress itself is also associated with sensory sensitivity [4, 7], it could be that in non-autistic individuals higher AQ scores were due to more psychological distress that also led to or was generated by higher sensory sensitivity, while in autistic individuals’ sensory sensitivity was associated with their autism and less influenced by psychological distress. On a subscale level, correlations between AQ and GSQ mostly persisted at least in the total sample and the autism group and even when controlling for psychological distress, which is in line with findings from the original English, French and Japanese validations [48, 50, 53]. There were only a few correlations in the non-autism group between AQ and GSQ on a subscale level, which matched the results found by Zeisel et al. [65] in the high AQ group of the student sample. This is very interesting in the sense that AQ and GSQ mean scores of their high AQ group were very similar to the scores of our autism group, but findings are closer to our non-autism group, suggesting that autism-like traits are differently associated with sensory sensitivity in autistic individuals than in non-autistic individuals with many autism-like traits.

As expected, hypo- and hypersensitivity were strongly associated, again with the correlation found for the non-autism group (0.62) being very similar to the high AQ group of the student sample of Zeisel et al. ([65], 0.63). In line with Kuiper et al. [35] and Sapey-Triomphe et al. [50], there were small to strong correlations between hypo- and hypersensitivity scores in different modalities especially in the autism group in the current study, suggesting that autistic more than non-autistic individuals can experience both hypo- as well as hypersensitivity in one modality. While Sapey-Triomphe et al. [50] found that these correlations differed between groups in the visual and vestibular modality and Kuiper et al. [35] found no significant differences between groups, here a difference in the auditory modality was found. The findings could be attributed to cultural differences, however, whether that is the case or whether the specific samples caused these differing results, should be explored in further studies ideally with larger samples. Confirming the sensory profiles of Sapey-Triomphe et al. [50] and contrary to Kuiper et al. [35] there were stronger correlations between the GSQ subscale scores in the autism group than in the non-autism group, suggesting that autistic individuals often experience sensory atypicalities in multiple sensory modalities, while non-autistic individuals do not.

Exploratory analyses showed more sensory sensitivity for women, which is in line with previous literature [13, 42]. However, there were no between-group differences. Similarly, there were no between-group differences regarding age. However, hyposensitivity increased with age, which could be related to the reduced sensory acuity with increased age which has previously been reported (e.g. [13]).

We expected to find an internal structure of the GSQ comparable to the original English study [48], however data did not seem to fit the single factor model well. An exploratory second-order factor model, similar to the findings of Sapey-Triomphe et al. [50] did not fit better, nor did any other exploratory model. A exploratory factor analyses could replicated the one-factor solution of the original questionnaire for the autism group [48], which was most important, since the questionnaire is autism specific. Determining which aspects of sensory sensitivity are assessed by the GSQ, should be a priority for future studies. E.g. another possibility for future studies could be a four-factor structure with the aspects of sensory processing proposed by Dunn [20] – poor registration, sensory seeking, sensitivity to stimuli, and sensory avoiding rather than grouping by hypo- and hypersensitivity or the modalities.

Diagnostic capabilities of the GSQ were assessed by calculating a cut-off score for heightened sensory sensitivity. The 95th percentile of GSQ scores in the non-autism group was much higher in this sample (65.6) than in Kuiper et al. ([35], 56.6) and the percentage of autistic individuals above that score much lower (1/3 compared to 2/3). Even when moving the cut-off to the 90th percentile, which with a score of 57 was very close to the 95th percentile score of Kuiper et al. [35], only half of the autistic participants scored higher. This could also be attributed to generally lower GSQ scores due to Covid-19 especially in autistic individuals. While our findings indicate that scores above 57 or 65 are common in autistic individuals and the GSQ can be used to indicate heightened sensory sensitivity, it should not be used to screen for ASD.

Overall, the goal of this study, was reached with confirming almost all hypotheses. Findings that were not present in the German student sample [65] but in previous validation studies were found here. This suggests a better suitability of the sample for detecting such effects. Only the factor structure of the German GSQ was not confirmed, which fits into the contradicting findings of previous validations [48, 50, 65]. While a strength of this study was the relatively large sample of autistic individuals over a broad age range, a lot of autistic participants had co-existing psychological disorders while non-autistic participants did not, and autistic individuals with intellectual disabilities were excluded. It is also important to note that the study took place during the Covid-19 pandemic, in which individuals experienced more psychological distress [1] or symptoms seen as autism-like traits, like feeling uneasy in social gatherings. Future studies should use larger samples especially for analyzing the factor structure and consider rephrasing or exclusion of the GSQ items, that did not seem to be satisfactory, neither in the German version nor in other versions (especially Items 17 and 36, which are also very similar).

Nevertheless, this German version of the GSQ is a validated self-report questionnaire assessing sensory sensitivity in autistic adults. It could be used to assess sensory sensitivity in the diagnostic process of ASD, however, interpretation lies in the discretion of the diagnostician as clear cut-offs for heightened sensory sensitivity must be established in larger samples in the future. Furthermore, this questionnaire can be useful to evaluate sensory atypicalities in a specific individual, to support them in using any strengths based on sensory sensitivities and modify their environment, to be more suitable considering their sensory processing and needs. The GSQ has been adapted for children with two versions for parents [51] and one for children [40]. Translating and validating those versions in German, could be an asset in the diagnostic process and for further research by also allowing the inclusion of autistic children and autistic participants with intellectual disabilities and giving us the possibility to further understand sensory sensitivity in autistic individuals on the entire autism spectrum.

## Supplementary Information

Supplementary Material 1.

## Data Availability

No datasets were generated or analysed during the current study.

## Abbreviations

ADI-R: Autism Diagnostic Interview-Revised
ADOS-2: Autism Diagnostic Observation Schedule Second Edition
ANOVA: Analysis of variance
AQ: Autism Spectrum Quotient
ASD: Autism Spectrum Disorder
CFA: Confirmatory factor analysis
CITC: Corrected item-total correlations
CSTC: Corrected subscale-total correlations
DSM-5: Diagnostic and Statistical Manual of Mental Disorders, Fifth Edition
GFI: Goodness-of-fit index
GG: Greenhouse-Geisser
GSI: Global severity index
GSQ: Glasgow Sensory Questionnaire
ICD: International Statistical Classification of Diseases and Related Health Problem
IQ: Intelligence quotient
M: Mean
ML: Maximum likelihood
OCD: Obsessive-compulsive disorder
OSF: Open Science Framework
RMSEA: Root mean square error of approximation
SAD: Social anxiety disorder
SCL-90-R: Symptom Checklist-90-Revised
SD: Standard deviation
TUD: Technische Universität Dresden (Technical University Dresden)
ULS: Unweighted least squares
