# Supplementary material for: Validation of the German Glasgow Sensory Questionnaire in autistic adults
Source: BMC Psychiatry. 2025 Jan 31;25:86. doi: 10.1186/s12888-025-06504-0 (PMC11786423; doi:10.1186/s12888-025-06504-0)
Supplement: Supplementary file 1 — Supplementary Material 1. [file 12888_2025_6504_MOESM1_ESM.docx]

# Supplementary Material

## Reliability

**Table S1***Item-total correlations of each item in the entire sample and both groups*

|  | ES^a^ | ASD^b^ | N-ASD^c^ |  |  | ES^a^ | ASD^b^ | N-ASD^c^ |
| --- | --- | --- | --- | --- | --- | --- | --- | --- |
| Item 1 | .45 | .47 | **.29** |  | Item 22 | **.19** | .38 | **.03** |
| Item 2 | .42 | .40 | **.27** |  | Item 23 | .44 | .38 | .39 |
| Item 3 | .35 | .31 | **.06** |  | Item 24 | .56 | .60 | .50 |
| Item 4 | .46 | .55 | .40 |  | Item 25 | .68 | .75 | .47 |
| Item 5 | .43 | .50 | **.24** |  | Item 26 | .41 | .33 | .53 |
| Item 6 | .56 | .55 | .49 |  | Item 27 | .44 | .41 | .33 |
| Item 7 | .47 | .62 | **.24** |  | Item 28 | **.19** | .32 | **.19** |
| Item 8 | .53 | .59 | .42 |  | Item 29 | .47 | .43 | .37 |
| Item 9 | .37 | .33 | .41 |  | Item 30 | .49 | .47 | .40 |
| Item 10 | .53 | .57 | **.28** |  | Item 31 | .66 | .68 | .49 |
| Item 11 | .52 | .59 | **.29** |  | Item 32 | .58 | .57 | .57 |
| Item 12 | .41 | .34 | **.24** |  | Item 33 | .53 | .62 | .48 |
| Item 13 | .59 | .61 | .39 |  | Item 34 | .48 | .49 | **.28** |
| Item 14 | .53 | .50 | .38 |  | Item 35 | .34 | .32 | **.28** |
| Item 15 | .56 | .53 | .42 |  | Item 36 | **.06** | **.04** | **.25** |
| Item 16 | .46 | .43 | .36 |  | Item 37 | .46 | .46 | **.30** |
| Item 17 | **.19** | **.22** | **.22** |  | Item 38 | .45 | .44 | **.28** |
| Item 18 | .49 | .48 | .41 |  | Item 39 | **.25** | **.19** | **.20** |
| Item 19 | .55 | .60 | .37 |  | Item 40 | .42 | .46 | **.29** |
| Item 20 | .54 | .46 | .46 |  | Item 41 | .40 | .37 | .37 |
| Item 21 | .56 | .68 | **.26** |  | Item 42 | .36 | .43 | **.24** |
|  | ES^a^ | ASD^b^ | N-ASD^c^ |  |  |  |  |  |
| *Mean* | .45 | .46 | .34 |  |  |  |  |  |
| *SD* | .02 | .02 | .01 |  |  |  |  |  |

*Note.* Depicted are the corrected item-total correlations and the mean and standard deviation of all item-total correlations per group. Item-total correlations below .3 are depicted in bold, as they are deemed unsatisfactory (Nurosis, 1994).
^a^ Entire Sample, ^b^ Autism Group, ^c^ Non-Autism Group.

**Table S2***Subscale-total correlations of each subscale in the entire sample and both groups*

|  | Entire sample | Autism group | Non-autism group |
| --- | --- | --- | --- |
| Visual hyposensitivity | .64 | .69 | .52 |
| Auditory hyposensitivity | .68 | .66 | .66 |
| Gustatory hyposensitivity | .43 | .43 | .34 |
| Olfactory hyposensitivity | .39 | .49 | .33 |
| Tactile hyposensitivity | .64 | .63 | .50 |
| Vestibular hyposensitivity | .56 | .50 | .42 |
| Proprioception hyposensitivity | .58 | .61 | .31 |
| Visual hypersensitivity | .65 | .70 | .48 |
| Auditory hypersensitivity | .71 | .72 | .56 |
| Gustatory hypersensitivity | .56 | .48 | .57 |
| Olfactory hypersensitivity | .69 | .73 | .49 |
| Tactile hypersensitivity | .51 | .59 | .27 |
| Vestibular hypersensitivity | .58 | .66 | .61 |
| Proprioception hypersensitivity | .62 | .60 | .50 |
|  |  |  |  |
| *Mean* | .60 | .61 | .47 |
| *SD* | .10 | .10 | .12 |

*Note.* Depicted are the corrected subscale-total correlations and the mean and standard deviation of all subscale-total correlations per group.

Post-hoc analysis revealed that Cronbach's Alpha of the German GSQ on an item level, χ^2^(1, *N* = 163) = 8.97, *p =*.003, φ = 0.23, as well as item-total correlations, *t*(82) = 4.48, *p*< .001, *d*= 0.99, were significantly greater in the autism group.

Cronbach's Alpha of the German GSQ on a subscale level, χ^2^(1, *N* = 163) = 6.88, *p =*.009, φ = 0.21, and corrected-subscale total correlations, *t*(26) = 3.41, *p*= .002, *d*= 1.34, were significantly greater in the autism group than in the non-autism group.

### Reliability as assessed with Guttmann split-half coefficient

To calculate Guttman split-half coefficient on an item level, odd items were assigned to the first and even items to the second half. The split-half coefficient on an item level was .94 in the entire sample (*N* = 163), .94 in the autism (*n =*80) and .89 in the non-autism group (*n =*83). To calculate Guttman Split-Half coefficient on a subscale level, the seven hyposensitivity subscales were assigned to the first and the seven hypersensitivity subscales to the second half. The Split-Half coefficient on a subscale level was .86 in the entire sample, .87 in the autism and .75 in the non-autism group.

## Comparability between the original and the German GSQ

Differences between total and item scores between this study and the original British study by Robertson & Simmons (2013) were assessed with two-sided independent sample *t-*tests (this was not preregistered). Python (3.10.09) was used to compare item scores of the original English GSQ (Robertson & Simmons, 2013) and the German version.

Mean total AQ score (*M* = 25.0, *SD*= 11.1, *N* = 172) was higher in this study than in the original study by Robertson & Simmons (2013, *M*= 22.5, *SD* = 10.6, *N* = 212), *t*(382) = 2.31, *p*= 0.02, *d*= 0.24. The mean total GSQ score (*M* = 46.7, *SD* = 21.8, *N* = 172) was lower than in the original study (*M* = 56.7, *SD* = 23.6, *N* = 212), *t*(382) = ‑4.27, *p*< 0.001, *d*= -0.44. There was a strong correlation between the GSQ mean item scores of this study and the study by Robertson & Simmons (2013), *r* = .88, *p* < .001, and all item mean scores were in the *M* ± *SD* interval of the item scores by Robertson & Simmons (2013, see Figure S1). However, when comparing the items of the different samples with *t‑*tests, there were differences between item scores in 18 of the items (see Table S3).

Comparing the sample of this study to the ones in the original British and the French studies it is surprising, that while the mean AQ score of this study (*M*= 25.0) was higher than the one in the original English (*M*= 22.5) and the French version (*M*= 23.0), the mean GSQ score was lower (*M*= 46.7, English: *M*= 56.7, French: *M*= 58.7, Robertson & Simmons, 2013; Sapey-Triomphe et al., 2018). It is interesting to note, that mean AQ and GSQ scores of the autism and the non-autism group of this study were very similar to the scores of the high and low AQ group respectively of the German student sample (Zeisel et al., 2023). The autism group and the non-autism group differed in their quantity of autism-like traits and sensory sensitivity similarly to the autism and non-autism group in the Japanese validation study (Takayama et al., 2013) and the high and low AQ group of the German student sample validation study (Zeisel et al., 2023), indicating that the sample was suitable. They differed less than the low and high AQ group of the French validation study (Sapey-Triomphe et al., 2018) and the autism and non-autism group in the Dutch study (Kuiper et al., 2019). For all 18 items whose mean scores differed between this study and the original English version, the scores of this study were lower, which is somewhat puzzling due to the higher mean AQ score and the larger number of autistic participants included in this study. A possible explanation might be that autistic and non-autistic individuals had experienced fewer of the situations described in the GSQ especially regarding hypersensitivity or were less bothered by them prior to answering the questionnaire, as the study took place in the first year of Covid-19 and people might have been staying in the comfort of their own home more. However, correlation between items of this German and the English version was still very strong (*r*= .88) and similar to the correlation between the French and the English versions (*r* = .88, Sapey-Triomphe et al., 2018).

**Figure S1***Mean scores of each item in the German and English version*


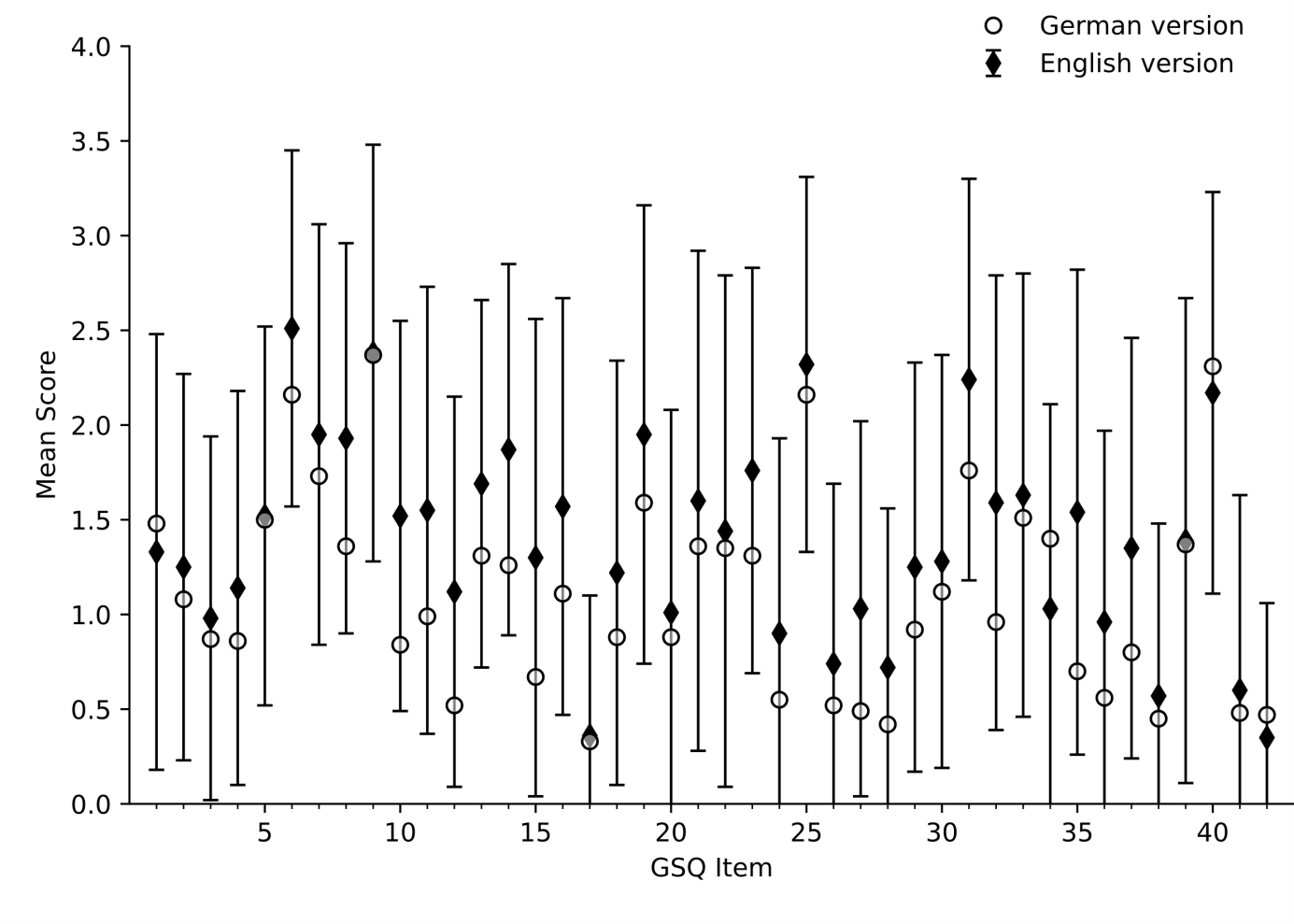
*Note.* Error bars show the standard deviations of each item in the English version.

**Table S3***Comparison between means and standard deviations of the original and the German GSQ*

|  | English  *N* = 212 | German  *N* = 172 | *p* |  |  | English  *N* = 212 | German  *N* = 172 | *p* |
| --- | --- | --- | --- | --- | --- | --- | --- | --- |
| Item 1 | 1.3 ± 1.2 | 1.5 ± 1.1 |  |  | Item 22 | 1.4 ± 1.4 | 1.4 ± 1.4 |  |
| Item 2 | 1.3 ± 1.0 | 1.1 ± 1.0 |  |  | Item 23 | 1.8 ± 1.1 | 1.3 ± 1.1 | .002** |
| Item 3 | 1.0 ± 1.0 | 0.9 ± 0.9 |  |  | Item 24 | 0.9 ± 1.0 | 0.6 ± 1.0 | .02* |
| Item 4 | 1.1 ± 1.0 | 0.9 ± 1.0 | .15 |  | Item 25 | 2.3 ± 1.0 | 2.2 ± 1.1 |  |
| Item 5 | 1.5 ± 1.0 | 1.5 ± 1.1 |  |  | Item 26 | 0.7 ± 1.0 | 0.5 ± 0.9 | .36 |
| Item 6 | 2.5 ± 0.9 | 2.2 ± 1.1 | .02* |  | Item 27 | 1.0 ± 1.0 | 0.5 ± 0.8 | <.001*** |
| Item 7 | 2.0 ± 1.1 | 1.7 ± 1.2 |  |  | Item 28 | 0.7 ± 0.8 | 0.4 ± 0.8 | .009** |
| Item 8 | 1.9 ± 1.0 | 1.4 ± 1.1 | <.001*** |  | Item 29 | 1.3 ± 1.1 | 0.9 ± 1.1 | .07 |
| Item 9 | 2.4 ± 1.1 | 2.4 ± 1.2 |  |  | Item 30 | 1.3 ± 1.1 | 1.1 ± 1.0 |  |
| Item 10 | 1.5 ± 1.0 | 0.8 ± 1.0 | <.001*** |  | Item 31 | 2.2 ± 1.1 | 1.8 ± 1.2 | .002** |
| Item 11 | 1.6 ± 1.2 | 1.0 ± 1.2 | <.001*** |  | Item 32 | 1.6 ± 1.2 | 1.0 ± 1.1 | <.001*** |
| Item 12 | 1.1 ± 1.0 | 0.5 ± 0.9 | <.001*** |  | Item 33 | 1.6 ± 1.2 | 1.5 ± 1.2 |  |
| Item 13 | 1.7 ± 1.0 | 1.3 ± 1.1 | .009** |  | Item 34 | 1.0 ± 1.1 | 1.4 ± 1.4 | .07 |
| Item 14 | 1.9 ± 1.0 | 1.3 ± 1.0 | <.001*** |  | Item 35 | 1.5 ± 1.3 | 0.7 ± 1.0 | <.001*** |
| Item 15 | 1.3 ± 1.3 | 0.7 ± 1.1 | <.001*** |  | Item 36 | 1.0 ± 1.0 | 0.6 ± 0.9 | .002** |
| Item 16 | 1.6 ± 1.1 | 1.1 ± 1.0 | .001** |  | Item 37 | 1.4 ± 1.1 | 0.8 ± 1.0 | <.001*** |
| Item 17 | 0.4 ± 0.7 | 0.3 ± 0.7 |  |  | Item 38 | 0.6 ± 0.9 | 0.5 ± 0.7 |  |
| Item 18 | 1.2 ± 1.1 | 0.9 ± 1.0 | .06 |  | Item 39 | 1.4 ± 1.3 | 1.4 ± 1.2 |  |
| Item 19 | 2.0 ± 1.2 | 1.6 ± 1.3 | .10 |  | Item 40 | 2.2 ± 1.1 | 2.3 ± 1.0 |  |
| Item 20 | 1.0 ± 1.1 | 0.9 ± 1.1 |  |  | Item 41 | 0.6 ± 1.0 | 0.5 ± 1.0 |  |
| Item 21 | 1.6 ± 1.3 | 1.4 ± 1.3 |  |  | Item 42 | 0.4 ± 0.7 | 0.5 ± 0.8 |  |

*Note*. Depicted are the means and standard deviations (*M* ± *SD*) for each item of the GSQ in the original English version (Robertson & Simmons, 2013) and in this sample of the German version and the level of significance of the difference between them, calculated with two-sided independent sample t-tests. Bonferroni-Holm correction applied to all *p*-values.
* significant on a .05 level ** significant on a .01 level *** significant on a .001 level
*p*-values of >.999 are not shown in the table.

## Main and interaction effects of the group x domain x modality ANOVA

**Table S4***Subscale scores of the GSQ in the autism and non-autism group (main effect of group)*

|  | Autism Group  (*n* = 86) | |  | Non-autism Group  (*n* = 86) | |  | Comparison | | | |  |
| --- | --- | --- | --- | --- | --- | --- | --- | --- | --- | --- | --- |
|  | *M* | *SD* |  | *M* | *SD* |  | *t* | df | *p* | *d* | |
| GSQ subscale scores | | |  |  |  |  |  |  |  |  | |
| - visual hypo^a^  - visual hyper^b^  - auditory hypo  - auditory hyper  - gustatory hypo  - gustatory hyper  - olfactory hypo  - olfactory hyper  - tactile hypo  - tactile hyper  - vestibular hypo  - vestibular hyper  - proprio^c^ hypo  - proprio hyper | 3.3  3.8  5.8  7.2  2.9  3.5  2.6  3.9  4.5  4.0  4.0  3.6  4.0  2.3 | 2.5  2.8  2.8  3.1  2.3  2.6  1.9  3.2  2.2  3.0  3.1  2.7  2.2  2.2 |  | 2.5  2.6  4.6  4.9  2.2  2.3  2.7  2.6  3.3  3.1  1.6  2.3  2.5  1.2 | 1.8  2.2  1.9  2.5  1.8  1.6  1.9  1.9  1.5  2.2  1.7  1.8  1.7  1.4 |  | 2.42  3.13  3.34  5.31  2.46  3.79  -0.36  3.27  4.23  2.23  6.26  3.63  5.05  3.96 | 155.6  159.9  148.72  160.1  170  144.4  170  138.7  150.9  155.1  131.3  149.6  155.7  155.6 | .06  .01*  .006**  < .001***  .06  .002 **  .72  .008**  < .001***  .06  < .001***  .003**  < .001***  .001** | 0.37  0.48  0.52  0.81  0.38  0.58  -0.05  0.50  0.65  0.34  0.96  0.55  0.78  0.60 | |

*Note*. Comparison between groups calculated with two-sided independent sample t-tests.
^a^ hypo = hyposensitivity, ^b^ hyper = hypersensitivity, ^c^ proprio = proprioception.
Bonferroni-Holm correction applied to all *p*-values.
* significant on a .05 level ** significant on a .01 level *** significant on a .001 level

**Table S5***Differences between modality scores for the entire sample (main effect of modality)*

|  | Auditory | Gustatory | Olfactory | Tactile | Vestibular | Proprio^a^ |
| --- | --- | --- | --- | --- | --- | --- |
| Visual  *M* = 6.1 *SD* = 4.2 | **df = 168,  *t*= -17.69, *p* < .001**  ***d* = -1.36** | df = 169,  *t*= 2.41, *p* = .1  *d* = 0.19 | df = 167,  *t*= 0.97, *p* > .99  *d* = 0.08 | **df = 169,  *t*= -4.32, *p* < .001**  ***d* = -0.33** | df = 168,  *t*= 1.94, *p* = .3  *d* = 0.15 | **df = 166,  *t*= 4.07, *p* < .001**  ***d* = 0.32** |
| Auditory  *M* = 11.2 *SD* = 4.8 |  | **df = 170,  *t*= 17.51, *p* < .001**  ***d* = 1.34** | **df = 168,  *t*= 18.21, *p* < .001**  ***d* = 1.40** | **df = 170,  *t*= 11.59, *p* < .001**  ***d* = 0.89** | **df = 169,  *t*= 20.45, *p* < .001**  ***d* = 1.57** | **df = 167,  *t*= 18.58, *p* < .001**  ***d* = 1.43** |
| Gustatory  *M* = 5.4 *SD* = 3.4 |  |  | df = 169,  *t*= -1.48, *p* = .6  *d* = -0.11 | **df = 171,  *t*= -7.27, *p* < .001**  ***d* = -0.55** | df = 170,  *t*= -0.87, *p* > .99  *d* = -0.07 | df = 168,  *t*= 1.75, *p* = .4  *d* = 0.14 |
| Olfactory  *M* = 5.9 *SD* = 3.7 |  |  |  | **df = 169,  *t*= -5.89, *p* < .001**  ***d* = -0.45** | df = 168,  *t*= 0.51, *p* > .99  *d* = 0.04 | **df = 166,  *t*= 3.18, *p* = .02**  ***d* = 0.25** |
| Tactile  *M* = 7.4 *SD* = 3.8 |  |  |  |  | **df = 170,  *t*= 5.65, *p* < .001**  ***d* = 0.43** | **df = 168,  *t*= 9.00, *p* < .001**  ***d* = 0.69** |
| Vestibular  *M* = 5.7 *SD* = 4.2 |  |  |  |  |  | df = 167,  *t*= 2.72, *p* = .06  *d* = 0.21 |
| Proprio  *M* = 4.9 *SD* = 3.5 |  |  |  |  |  |  |

*Note.* Depicted are the results of paired *t*-tests.
^a^ Proprioception
Bonferroni-Holm correction applied to all *p-*values. Significant comparisons are depicted in bold.

**Figure S2***GSQ modality scores in the autism and the non-autism group (interaction effect group x modality)*

*
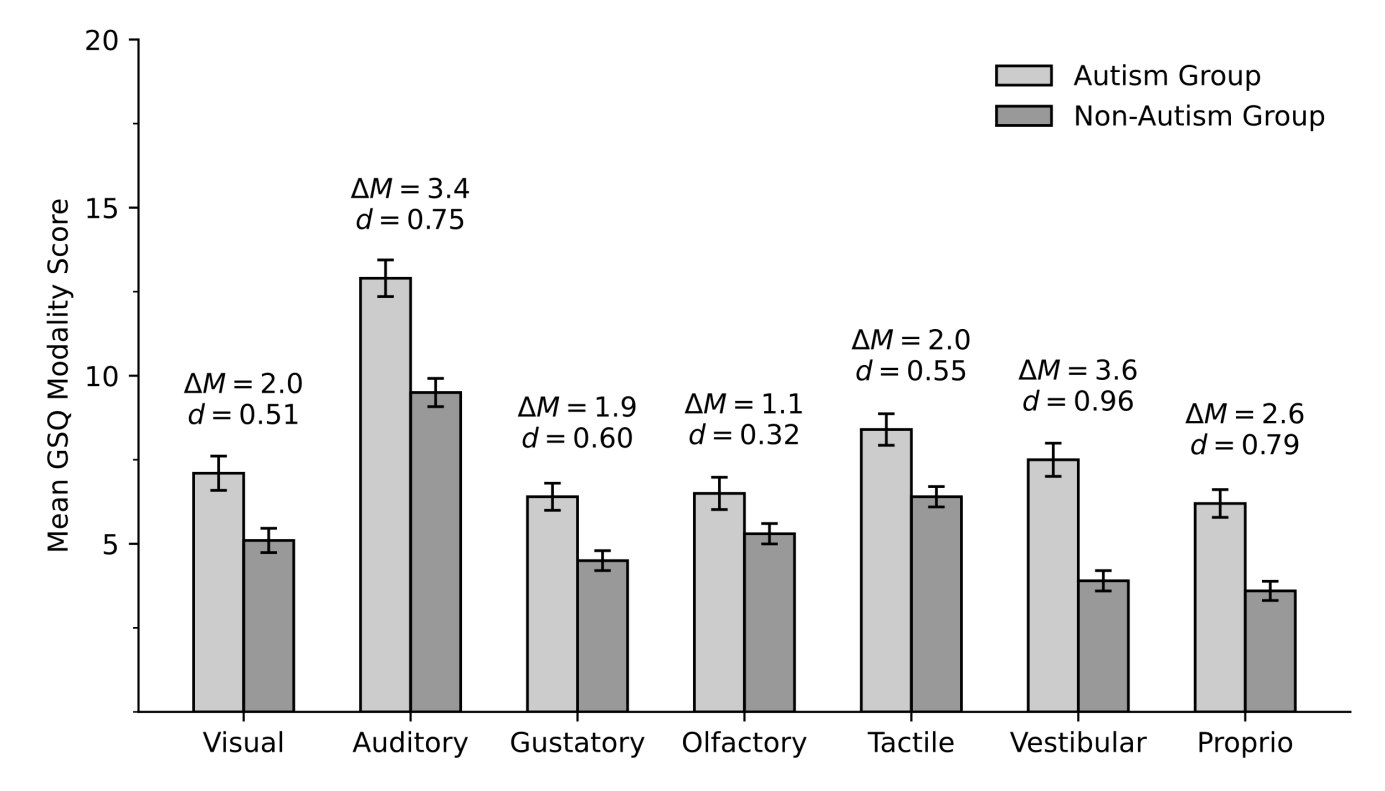
*

*Note.* Depicted are the mean scores of each modality in the autism group and the non-autism group and the differences between the mean scores of the autism and the non-autism group (Δ*M*). Error bars show the standard errors. Proprio = Proprioception
All differences are significant (see Table 3).

**Table S6***GSQ modality scores for the domains hypo- and hypersensitivity in the entire sample and both groups (interaction effects modality x domain and group x modality x domain)*

|  | Hyposensitivity | | |  | Hypersensitivity | |  | Comparison | | | |  |
| --- | --- | --- | --- | --- | --- | --- | --- | --- | --- | --- | --- | --- |
|  | *M* | *SD* | |  | *M* | *SD* |  | *t* | df | *p* | *d* |  |
| **Entire sample** |  | | |  |  | |  |  |  |  |  |  |
| Visual | 2.9 | 2.3 | |  | 3.2 | 2.6 |  | -1.50 | 169 | .3 | -0.11 |  |
| Auditory | 5.2 | 2.5 | |  | 6.1 | 3.0 |  | -4.72 | 170 | **<.001** | -0.36 |  |
| Gustatory | 2.5 | 2.1 | |  | 2.9 | 2.2 |  | -1.90 | 171 | .2 | -0.14 |  |
| Olfactory | 2.6 | 1.9 | |  | 3.2 | 2.7 |  | -2.66 | 169 | **.04** | -0.20 |  |
| Tactile | 3.9 | 2.0 | |  | 3.5 | 2.6 |  | 2.07 | 171 | .2 | 0.16 |  |
| Vestibular | 2.8 | 2.8 | |  | 2.9 | 2.4 |  | -0.31 | 170 | .8 | -0.02 |  |
| Proprioception | 3.2 | 2.1 | |  | 1.7 | 1.9 |  | 10.01 | 168 | **<.001** | 0.77 |  |
| **Autism Group** |  |  | |  |  |  |  |  |  |  |  |  |
| Visual | 3.3 | 2.5 | |  | 3.8 | 2.8 |  | -1.80 | 84 | .2 | -0.20 |  |
| Auditory | 5.8 | 2.8 | |  | 7.2 | 3.1 |  | -4.83 | 84 | **<.001** | -0.52 |  |
| Gustatory | 2.9 | 2.3 | |  | 3.5 | 2.6 |  | -1.90 | 85 | .2 | -0.21 |  |
| Olfactory | 2.6 | 1.9 | |  | 3.9 | 3.2 |  | -4.15 | 83 | **<.001** | -0.45 |  |
| Tactile | 4.5 | 2.2 | |  | 4.0 | 3.0 |  | 1.96 | 85 | .2 | 0.21 |  |
| Vestibular | 4.0 | 3.1 | |  | 3.6 | 2.7 |  | 1.21 | 85 | .2 | 0.13 |  |
| Proprioception | 4.0 | 2.2 | |  | 2.3 | 2.2 |  | 7.49 | 83 | **<.001** | 0.82 |  |
| **Non-autism group** | | |  |  |  |  |  |  |  |  |  | |
| Visual | 2.5 | 1.8 | |  | 2.6 | 2.2 |  | -0.24 | 84 | >.999 | -0.03 |  |
| Auditory | 4.6 | 1.9 | |  | 4.9 | 2.5 |  | -1.64 | 85 | .5 | -0.18 |  |
| Gustatory | 2.2 | 1.8 | |  | 2.3 | 1.6 |  | -0.58 | 85 | >.999 | -0.06 |  |
| Olfactory | 2.7 | 1.9 | |  | 2.6 | 2.0 |  | 0.40 | 85 | >.999 | 0.04 |  |
| Tactile | 3.3 | 1.5 | |  | 3.1 | 2.2 |  | 0.92 | 85 | >.999 | 0.10 |  |
| Vestibular | 1.6 | 1.7 | |  | 2.3 | 1.8 |  | -2.59 | 84 | .07 | -0.28 |  |
| Proprioception | 2.5 | 1.7 | |  | 1.2 | 1.4 |  | 6.76 | 84 | **<.001** | 0.73 |  |

*Note.* Depicted are the means and standard deviations of each modality in the domains hypo- and hyper­sensitivity for the entire sample, the autism group and the non-autism group and the results of paired *t*-tests. Bonferroni-Holm correction applied to all *p-*values (for each group). Significant comparisons depicted in bold.

The post-hoc tests revealed that in the entire sample as well as in the autism group more hyper- than hyposensitivity was reported in the auditory and olfactory modality and more hypo- than hypersensitivity was reported regarding proprioception. Non-autistic individuals only reported more hypo- than hypersensitivity regarding proprioception but no differences in the auditory or olfactory modality.

## Further exploratory analyses regarding sex and age

Since there are studies showing higher sensory sensitivity for autistic females (Carlton et al., 2024; Osório et al., 2021) as well as studies indicating reduced sensory acuity with age (Carlton et al., 2024), we exploratorily investigated the effects of age and sex on sensory sensitivity. There was no significant correlation between total GSQ score and age in the entire sample, *r* = .06, *p* = .46, the autism group, *r* = .12, *p* = .29, or the non-autism group, *r* = .02, *p* = .86. There was also no significant difference between males and females in the total GSQ score in the entire sample, *t*(170) = -1.77, *p* = .08 (males *M* = 45.1, *SD* = 21.2; females *M* = 52.0, *SD* = 23.1), the autism group, *t*(84) = -1.51, *p* = .14 (males *M* = 52.9, *SD* = 23.8; females *M* = 62.1, *SD* = 25.1) or the non-autism group, *t*(84) = -1.05, *p* = .30 (males *M* = 37.4, *SD* = 14.7; females *M* = 41.3, *SD* = 14.9).

For further analysis we used a 2 (*group* [autism, non-autism]) x 2 (sex [male, female] x 2 (*domain* [hypo, hyper]) mixed ANOVA with age as a covariate. There was a *domain* x *age* interaction effect, *F*(1, 158.0) = 6.67 (GG), *p* = .01, a *domain* x *gender* interaction effect, *F*(1, 158.0) = 16.04 (GG), *p* < .001, a *modality* x *gender* interaction effect, *F*(5.0, 797.1) = 9.52 (GG), *p* = .04 and a *domain* x *modality* x *age* interaction effect, *F*(5.5, 871.2) = 3.22 (GG), *p* = .005.

Post-hoc t-tests revealed that female participants reported significantly more sensory sensitivity in the domain hypersensitivity, *t*(170) = -1.78, *p* = .005 (males *M* = 22.1, females *M* = 28.4), in the modalities tactile, *t*(170) = -2.44, *p* = .02 (males *M* = 7.0, females *M* = 8.6), and vestibular, *t*(169) = -2.21, *p* = .03 (males *M* = 5.3, females *M* = 7.0), and in the subscales visual hypersensitivity, *t*(52.5) = -2.11, *p* = .004 (males *M* = 3.0, females *M* = 4.1), tactile hypersensitivity, *t*(170) = -3.66, *p* < .001 (males *M* = 3.1, females *M* = 4.8), and vestibular hypersensitivity, *t*(170) = -3.17, *p* = .002 (males *M* = 2.6, females *M* = 3.9).

Post-hoc tests revealed a strong positive correlation between age and hyposensitivity, *r* = .73, *p* < .001, but no significant correlation between age and hypersensitivity, *r* = .12, *p* = .1. There were no significant correlations between age and modality scores, but three small but positive correlations on a subscale level. There were correlations between age and tactile hypersensitivity, *r* = .18, *p* = .02, vestibular hypersensitivity, *r* = .17, *p* = .02, and proprioception hyposensitivity, *r* = .17, *p* = .03.

## Association between AQ and GSQ subscales

Associations between psychological distress and autism-like traits as well as sensory sensitivity: There was a moderate positive correlation between SCL-90-R GSI T-value and AQ in the entire sample, r = .47, *p* < .001, a non-significant small positive correlation in the autism group, r = .20, *p* = .06, and a strong positive correlation in the non-autism group, r = .52, *p* < .001. SCL-90-R GSI T-value and GSQ correlated strongly (entire sample: r = .55, *p* < .001, autism group: r = .51, *p* < .001, non-autism group: r = .51, *p* < .001).

Correlations between AQ total/ subscale scores and GSQ total/ domain scores as well as between AQ total and GSQ modality and subscale scores are presented in Tables S7 and S8. There were strong correlations between AQ and GSQ domains in the entire sample and the autism group and moderate correlations in the non-autism group. When controlling for psychological distress, moderate correlations remained in the entire sample and the autism group, but not in the non-autism group. There were moderate to strong correlations between AQ subscales and GSQ in the entire sample and small (subscale imagination) to strong correlations in the autism group, with a strong correlation of the AQ subscale attention to detail with hyposensitivity, but only a small correlation with hypersensitivity. While most correlations were lower when controlling for psychological distress, all remained significant. In the non-autism group only three of the AQ subscales correlated with the GSQ and only one with hypo- and two with hypersensitivity. Controlling for psychological distress only the moderate correlation between the subscale attention to detail and hyposensitivity remained. There were moderate to strong correlations between the GSQ modalities and AQ in the entire sample, small (gustatory) to strong (auditory) correlations in the autism group and small to moderate correlations for four modalities in the non-autism group (no significant correlations between AQ and the modalities gustatory, olfactory and proprioception), which mostly remained consistent when controlling for psychological distress (the correlation between the visual modality and AQ did not remain significant in the non-autism group). There were small to strong correlations between the GSQ subscales and AQ in the entire sample and the autism group, except for a non-significant correlation between olfactory hyposensitivity and AQ in the entire sample and a non-significant correlation between gustatory hypersensitivity and AQ in the autism group. When controlling for psychological distress the correlation between gustatory hyposensitivity and AQ in the entire sample and the correlation between proprioception hypersensitivity and AQ in the autism group became non-significant, while all others remained. In the non-autism group only five out of the 14 GSQ subscales correlated with total AQ (auditory and vestibular hypo, visual, auditory and vestibular hyper). None of the correlations remained significant when controlling for psychological distress.

**Table S7***Correlation between AQ total and subscale and GSQ total and domain scores in the entire sample, the autism and the non-autism group*

|  | Entire sample | |  | Autism group | | | |  | | | Non-autism group | | | | | | |  |
| --- | --- | --- | --- | --- | --- | --- | --- | --- | --- | --- | --- | --- | --- | --- | --- | --- | --- | --- |
|  | *r*^a^ | Contr.^b^ |  | *r^a^* | Contr.^b^ | |  | | | *r^a^* | | | Contr.^b^ | | | | |  |
|  | **GSQ total** | | | | | | | | | | | | | | | | | |
| AQ total | .60** | .46** |  | .55** | | .53** | |  | | | .42** | | | .20 | |  |  |  |
| AQ subscales  - social skill  - attention switching  - attention to detail  - communication  - imagination | .48**  .52**  .41**  .59**  .34** | .35**  .37**  .34**  .43**  .24** |  | .41**  .40**  .38**  .50**  .25* | | .47**  .34**  .35**  .44**  .28* | |  | | | .17  .37**  .31*  .41**  .09 | | | -.02  .24  .24  .18  -.03 | |  |  |  |
|  | **GSQ domain hyposensitivity** | | | | | | | | | | | | | | | | | |
| AQ total | .56** | .41** |  | .52** | | .49** | | |  | | | .31* | | | .08 | |  |  |
| AQ subscales  - social skill  - attention switching  - attention to detail  - communication  - imagination | .37**  .45**  .50**  .53**  .32** | .23**  .30**  .44**  .37**  .22** |  | .30*  .35**  .50**  .47**  .23* | | .33**  .29*  .48**  .40**  .26* | | |  | | | .03  .25  .38**  .27  .07 | | | -.18  .09  .33*  .01  -.04 | |  |  |
|  | **GSQ domain hypersensitivity** | | | | | | | | | | | | | | | | | |
| AQ total | .58** | .44** |  | .52** | | .49** | |  | | | .43** | | | .26 | |  |  |  |
| AQ subscales  - social skill  - attention switching  - attention to detail  - communication  - imagination | .50**  .52**  .30**  .57**  .33** | .39**  .37**  .20**  .42**  .22** |  | .45**  .40**  .25*  .48**  .24* | | .52**  .34**  .20*  .41**  .27* | |  | | | .26  .41**  .19  .45**  .09 | | | .12  .30  .11  .28  -.01 | |  |  |  |

*Note.* Depicted are the Pearson correlations (one-sided) between AQ total/ subscale scores and GSQ total/ domain scores as well as results of partial correlation analyses, controlling for SCL-90-R GSI T-value.
^a^ results of one-sides Pearson correlations, ^b^ results of one-sided partial correlation analyses.
Bonferroni-Holm correction applied to all *p*-values (per group, separately for Pearson correlation and partial correlation).
* significant on a .05 level ** significant on a .01 level

**Table S8***Correlation between AQ total and GSQ modality and subscale scores in the entire sample, the autism and the non-autism group*

|  | AQ total | | | | | | | |  |  |
| --- | --- | --- | --- | --- | --- | --- | --- | --- | --- | --- |
|  | Entire Sample | |  | Autism Group | |  | Non-autism Group | | |  |
|  | *r*^a^ | Contr.^b^ |  | *r*^a^ | Contr.^b^ |  | *r*^a^ | Contr.^b^ | |  |
| GSQ modalities  - visual  - auditory  - gustatory  - olfactory  - tactile  - vestibular  - proprioception | .47**  .48**  .34**  .36**  .46**  .60**  .49** | .35**  .46**  .22**  .28**  .37**  .50**  .35** |  | .48**  .52**  .25**  .46**  .45**  .46**  .43** | .49**  .50**  .21*  .46**  .46**  .39**  .38** |  | .32**  .45**  .11  .15  .29*  .47**  .20 | .14  .34**  -.02  .02  .12  .35**  -.01 | |  |
| GSQ subscales  - visual hypo^c^  - auditory hypo  - gustatory hypo  - olfactory hypo  - tactile hypo  - vestibular hypo  - proprioception hypo | .36**  .44**  .46**  .11  .42**  .54**  .48** | .25**  .33**  .16  .06  .30**  .45**  .35** |  | .40**  .37**  .28*  .35**  .33**  .38**  .47** | .38**  .36**  .26*  .33**  .33**  .36**  .47** |  | .22  .40**  .04  -.04  .26  .38**  .13 | .09  .30  -.09  -.15  .06  .27  -.11 | |  |
| - visual hyper^d^  - auditory hyper  - gustatory hyper  - olfactory hyper  - tactile hyper  - vestibular hyper  - proprioception hyper | .44**  .59**  .29**  .43**  .37**  .45**  .36** | .35**  .47**  .18*  .34**  .31**  .33**  .23** |  | .45**  .55**  .14  .44**  .44**  .38**  .23* | .48**  .52**  .09  .42**  .43**  .36**  .18 |  | .31*  .40**  .14  .25  .20  .37**  .22 | .14  .30  .07  .16  .11  .26  .11 | |  |

*Note.* Depicted are the Pearson correlations (one-sided) between AQ total/ subscale scores and GSQ total/ domain scores as well as results of partial correlation analyses, controlling for SCL-90-R GSI T-value.
^a^ results of one-sides Pearson correlations, ^b^ results of one-sided partial correlation analyses,
^c^ hyposensitivity, ^d^ hypersensitivity.
Bonferroni-Holm correction applied to all *p*-values (per group and separately for modalities and subscales and Pearson correlation and partial correlation).
* significant on a .05 level ** significant on a .01 level

## Factor structure of the GSQ

**Figure S3***Single Factor model of the GSQ*

**Table S9***Standardized regression coefficients of each item in the entire sample and both groups*

|  | ES^a^ | ASD^b^ | N-ASD^c^ |  |  | ES^a^ | ASD^b^ | N-ASD^c^ |
| --- | --- | --- | --- | --- | --- | --- | --- | --- |
| Item 1 | .45 | .49 | **.28** |  | Item 22 | **.17** | .41 | **.01** |
| Item 2 | .41 | .41 | **.24** |  | Item 23 | .49 | .40 | .39 |
| Item 3 | .35 | .32 | **-.003** |  | Item 24 | .58 | .64 | .45 |
| Item 4 | .50 | .56 | .45 |  | Item 25 | .72 | .80 | .58 |
| Item 5 | .37 | .52 | **.16** |  | Item 26 | .43 | .34 | .52 |
| Item 6 | .59 | .59 | .59 |  | Item 27 | .43 | .39 | .31 |
| Item 7 | .47 | .65 | **.21** |  | Item 28 | **.18** | .34 | **.17** |
| Item 8 | .55 | .61 | .55 |  | Item 29 | .46 | .44 | .34 |
| Item 9 | .46 | .37 | .51 |  | Item 30 | .54 | .48 | .45 |
| Item 10 | .55 | .60 | .38 |  | Item 31 | .68 | .70 | .58 |
| Item 11 | .56 | .61 | .39 |  | Item 32 | .61 | .59 | .59 |
| Item 12 | .46 | .35 | **.20** |  | Item 33 | .56 | .65 | .61 |
| Item 13 | .61 | .65 | .41 |  | Item 34 | .53 | .51 | .41 |
| Item 14 | .52 | .52 | .32 |  | Item 35 | .34 | .30 | **.23** |
| Item 15 | .58 | .55 | .38 |  | Item 36 | **.05** | **.02** | **.15** |
| Item 16 | .43 | .42 | .30 |  | Item 37 | .45 | .46 | **.24** |
| Item 17 | **.12** | **.22** | **.16** |  | Item 38 | .44 | .45 | **.19** |
| Item 18 | .48 | .49 | .42 |  | Item 39 | **.20** | **.17** | **.14** |
| Item 19 | .54 | .62 | .43 |  | Item 40 | .43 | .49 | .38 |
| Item 20 | .59 | .48 | .48 |  | Item 41 | .39 | .36 | .43 |
| Item 21 | .58 | .71 | **.27** |  | Item 42 | .37 | .43 | **.25** |
|  | ES^a^ | ASD^b^ | N-ASD^c^ |  |  |  |  |  |
| *Mean* | .46 | .48 | .35 |  |  |  |  |  |
| *SD* | .15 | .15 | .16 |  |  |  |  |  |

*Note.* Depicted are the standardized regression coefficients of each item from the one-factor CFA with ULS method and the mean and standard deviation of all item-total regression coefficients per group. Standardized regression coefficients below .3 are depicted in bold.
^a^ Entire Sample, ^b^ Autism Group, ^c^ Non-Autism Group

**Figure S4***Second-Order Factor model of the GSQ*

**Figure S5***Third-Order Factor model of the GSQ*

*Note.* Proprio = Proprioception.

**Table S10***Fit indices of the exploratory confirmatory factor analyses*

|  | χ^2^ | df | *p* | χ^2^/*df ^a^* | GFI^b^ | CFI^c^ | RMSEA^d^ |
| --- | --- | --- | --- | --- | --- | --- | --- |
| **Exploratory confirmatory factor analyses with the exclusion of ill-fitting items** | | | | | | | |
| Entire Sample^1^  - ULS^e^  - ML^f^ | 813.8  1236.5 | 629  629 | *<*.001 | 1.29  1.97 | .94 | .69 | .078* |
| Autism Group^2^  - ULS  - ML | 1040.9  1251.6 | 702  702 | *<*.001 | 1.48  1.78 | .92 | .58 | .100* |
| Non-Autism Group^3^  - ULS  - ML | 350.6  872.6 | 527  527 | < .001 | 0.67  1.66 | .85 | .50 | .089* |
| **Exploratory confirmatory factor analyses of the second-order model of the GSQ** | | | | | | | |
| Entire Sample  - ULS^4^  - ML | 1009.9  1513.3 | 819  818 | *<*.001 | 1.23  1.85 | .93 | .66 | .073* |
| Autism Group  - ULS^5^  - ML^6^ | 1125.8  1395.6 | 819  819 | *<*.001 | 1.37  1.70 | .91 | .57 | .094* |
| Non-Autism Group  - ULS^7^  - ML | 552.0  1333.8 | 819  818 | < .001 | 0.67  1.63 | .81 | .42 | .088* |
| **Exploratory confirmatory factor analyses of the third-order model of the GSQ** | | | | | | | |
| Entire Sample  - ULS^8^  - ML^9^ | 899.1  1381.3 | 807  808 | *<*.001 | 1.11  1.71 | .94 | .72 | .067* |
| Autism Group  - ULS^10^  - ML^11^ | 1025.1  1293.0 | 807  807 | *<*.001 | 1.27  1.60 | .92 | .64 | .087* |
| Non-Autism Group  - ULS^12^  - ML^13^ | 532.3  1282.6 | 810  808 | < .001 | 0.66  1.59 | .82 | .46 | .085* |

*Note.* Depicted are the fit indices of all exploratory confirmatory factor analyses.

^a^ relative / normed chi-square, ^b^ Goodness-of-fit statistic, ^c^ Comparative fit index, ^d^ Root mean square error of approximation, ^e^ Unweighted least squares method, ^f^ Maximum Likelihood method.

^1^ exclusion of the Items 17, 22, 28, 36 and 39.
^2^ exclusion of the Items 17, 36 and 39 (item-total correlations and standardized regression weights below .3).
^3^ exclusion of the Items 3, 5, 17, 22, 28, 36, 38 and 39 (standardized regression weights below .2, all item-total correlations below .3 as well).
^4^  negative variance of hyposensitivity error (- 0.009) set to zero
^5^  negative variance of hyposensitivity error (- 0.03) set to zero
^6^  negative variance of hyposensitivity error (- 0.009) set to zero
^7^  negative variance of hyposensitivity error (- 0.001) set to zero
^8^  negative variances of hyposensitivity error (- 0.022), auditory hypo error (- 0.043) and gustatory hypo error (‑ .005) set to zero
^9^  negative variances of hyposensitivity error (- 0.013), proprioception hyper (- 0.012), auditory hypo error (‑ 0.063) and gustatory hypo error (- 0.003) set to zero
^10^  negative variances of hyposensitivity error (- 0.048), gustatory hypo error (- 0.017) and proprioception hypo error (- 0.003) set to zero
^11^  negative variances of hyposensitivity error (- 0.044), auditory hypo error (- 0.001) and gustatory hypo error (‑ 0.01) set to zero
^12^  negative variances of hyposensitivity error (- 0.005), vestibular hyper error (- 0.017), proprioception hyper (‑ 0.004), visual hypo error (- 0.053), auditory hypo error (- 0.09) and gustatory hypo error (- 0.004) set to zero
^13^  negative variances of vestibular hyper error (- 0.013), visual hypo error (- 0.021), auditory hypo error (- 0.102) and gustatory hypo error (- 0.002) set to zero
* significant on a .05 level
